# The International Medical Congress

**Published:** 1881-07

**Authors:** 


THE INTERNATIONAL MEDICAL CONGRESS.—August next will doubtless witness the largest and most imposing body of medical men that were ever assembled in London or any other part of the world. Great talent, learning and eminent standing will doubtless be found in larger numbers in that august assemblage than in any other great council that has ever convened on any occasion, or for any purpose within the historic period. And such being the case, we who may not be there, will naturally look forward to its deliberations and their results, with reasonable expectation of decided gains to our science, and consequently to humanity at large. The only real fear is, that in these days of rapid transit and cheap transportation, too large a delegation of *mediocre* men may find their way into the congress, and embarrass or consume valuable time that should be occupied by the great lights and thinkers of the age in which we live!

